# Editorial: Adherence to the Mediterranean diet: microbiota and non-communicable diseases

**DOI:** 10.3389/fnut.2023.1352672

**Published:** 2024-01-08

**Authors:** Mireille Serhan, Sofi G. Julien

**Affiliations:** ^1^Department of Nutritional Sciences, Faculty of Health Sciences, University of Balamand, Deir el Balamand, Lebanon; ^2^Department of Nutrition and Food Sciences, Faculty of Arts and Sciences, Holy Spirit University of Kaslik, Jounieh, Lebanon

**Keywords:** Mediterranean diet, non-communicable diseases, microbiota, dietary habit, lifestyle

Effects of diet have long been associated with non-communicable diseases, which have exhibited a dramatic worldwide increased prevalence within the last decade (1). Special attention has been given to the Mediterranean diet (MD) because of its protective effects against type 2 diabetes, cancer, inflammatory diseases, metabolic syndrome, liver diseases or cardiovascular diseases (CVD) (2, 3). Although that some associations have been well established, research on the causal influence of such dietary pattern on health outcomes yielded inconsistent findings because of the emerging role of dietary-based microbiota composition in the progression of the diseases (4). It is therefore important to gain more comprehensive understandings of the association between the levels of the adherence to the Mediterranean diet (AMD) and co-morbidities among various population based on their age and/or groups gender and/or ethnicity and their cross-talk with gut microbiome.

This Research Topic includes the contribution of 54 authors from 9 articles presenting studies made in China, Iran, Iraq, Luxembourg, Spain, United States and New Zealand and treating recent findings on the aspects covered above.

The work of Al Kudsee et al. evaluated the association between the Alternative healthy eating index (AHEI) and the Mediterranean diet score (MDS), and the odds of metabolic syndrome (MetS) from 1,404 adults of the nationwide cross-sectional ORISCAV-LUX2 study in Luxembourg used to assess CVDs and their risk factors. Interestingly, the study demonstrated that CVD components of the MetS and the continuous MetS severity score was inversely correlated with the two dietary indices AHEI and MDS. These results highlight the importance of high AMD as preventive measure against MetS.

Chen et al. investigated the associations of adherence to MD pattern with incident rosacea through a prospective cohort study of government employees in China, in the absence of literature regarding the role of diet on rosacea. Results suggested that adherence MD pattern might reduce the risk of incident rosacea among non-overweight individuals.

The study of Liu and Lu discussed the research patterns, existing state, and possible hotspots in implementing the MD for the prevention and treatment of cancer using bibliometrics between 2012 and 2021. Results stressed on the value of the MD as a good regimen for lowering the risk of cancer and suggested that more research focusing on molecular mechanisms and clinical studies are required since the topic is receiving continuously a special attention.

A community-based cohort study conducted by Yiannakou et al. examined the associations between adherence to four established MD indices and breast cancer risk in women in the Framingham Offspring Study. Higher adherence to the MD was associated with a lower risk of breast cancer incidence. Findings suggest that the methodology and the composition of MD indices influence their ability to assess conformity to this specific diet pattern and predict disease risk. Similarly, the work performed by Sadeghi et al. evaluated the association between adherence to a MD and breast cancer among 350 Iranian women. Results reveal a significant inverse association between the MD and breast cancer and that adherence to Mediterranean dietary pattern was associated with reduced odds of breast cancer.

The study done by Melguizo-Ibáñez, Zurita-Ortega et al. showed that male adolescents in Spain were more prone to adhere adequately to the MD in contrast to teenager female, who exhibited higher consumption of alcohol beverage. Such lifestyle habits in youth male contributed to a better quality of life. These findings may serve as reference to investigate further risk factor for NCD in this group population. Another study conducted by the same authors Melguizo-Ibáñez, González-Valero et al. examined the association between the adherence to MD and self-concept and anxiety as a function of weekly physical activity in higher education context. Results show that students having low adherence to MD had higher levels of anxiety compared to those having a high degree of adherence. The same group reported high scores in the different dimensions of self-concept. Findings show that active lifestyle demonstrates improvement in the effect of a healthy dietary pattern on the different dimensions of self-concept and anxiety.

Fateh et al. conducted a clinical randomized control trial and provided evidence that a 12-week intervention based on a 250 ml daily intake of beetroot from Iranian adults significantly potentize beneficial effect of MD on lipid profile by lowering levels of cholesterol, triglycerides and LDL and by decreasing specific liver enzymatic markers contributing to reduction of steatosis, a conditions of fatty liver diseases.

Lithander et al. tested whether an intervention including a Mediterranean dietary pattern incorporating high quality New Zealand foods (NZMedDiet pattern) and behavior change science can improve the metabolic health of 200 participants and their household. Authors have demonstrated that the approach of both a household/whanau based intervention and the provision of food for a whole diet intervention is acceptable and culturally relevant for people in Aotearoa New Zealand.

Altogether, the results presented in this Research Topic shed light on the importance of MD in the prevention of NCD and its potential impact on microbiota modulation. These findings contributed to the understanding of the influence of some components of the MD on NCD outcomes, and may serve as guidelines for nutritional intervention or as guide for future works on the field.

## Author contributions

MS: Writing – original draft, Writing – review & editing. SJ: Writing – original draft, Writing – review & editing.

## References

[1] Dominguez LJ Di Bella G Veronese N Barbagallo M. Impact of mediterranean diet on chronic non-communicable diseases and longevity. Nutrients. (2021) 13:2028. 10.3390/nu13062028 34204683 PMC8231595

[2] Cena H Calder PC. Defining a healthy diet: evidence for the role of contemporary dietary patterns in health and disease. Nutrients. (2020) 12:334. 10.3390/nu12020334 32012681 PMC7071223

[3] Chen Y Zhou J Wang L. Role and mechanism of gut microbiota in human disease. Front Cell Infect Microbiol. (2021) 11:625913. 10.3389/fcimb.2021.625913 33816335 PMC8010197

[4] Budreviciute A Damiati S Sabir DK Onder K Schuller-Goetzburg P Plakys G. Management and prevention strategies for non-communicable diseases (NCDs) and their risk factors. Front Public Health. (2020) 8:574111. 10.3389/fpubh.2020.574111 33324597 PMC7726193

